# Tuning Stoichiometry and Structure of Pd-WO_3−*x*_ Thin Films for Hydrogen Gas Sensing by High-Power Impulse Magnetron Sputtering

**DOI:** 10.3390/ma13225101

**Published:** 2020-11-12

**Authors:** Nirmal Kumar, Stanislav Haviar, Jiří Rezek, Pavel Baroch, Petr Zeman

**Affiliations:** Department of Physics and NTIS—European Centre of Excellence, Faculty of Applied Sciences, University of West Bohemia, Univerzitní 8, 306 14 Plzeň, Czech Republic; kumarn@kfy.zcu.cz (N.K.); jrezek@ntis.zcu.cz (J.R.); pbaroch@kfy.zcu.cz (P.B.); zemanp@kfy.zcu.cz (P.Z.)

**Keywords:** tungsten oxide, WO_3_, hydrogen sensing, conductometric gas sensor, HiPIMS, stoichiometry

## Abstract

By tuning the deposition parameters of reactive high-power impulse magnetron sputtering, specifically the pulse length, we were able to prepare WO_3−*x*_ films with various stoichiometry and structure. Subsequently, the films were annealed in air at moderate temperature (350 °C). We demonstrate that the stoichiometry of the as-deposited films influences considerably the type of crystalline phase formed in the annealed films. The appropriate sub-stoichiometry of the films (approx. WO_2.76_) enabled crystallization of the monoclinic phase during the annealing. This phase is favorable for hydrogen sensing applications. To characterize the sensory behavior of the films, the tungsten oxide films were decorated by Pd nanoparticles before annealing and were assembled as a conductometric gas sensor. The sensory response of the films that crystallized in the monoclinic structure was proven to be superior to that of the films containing other phases.

## 1. Introduction

Hydrogen is a reliable and prospective source of energy because of its clean and renewable essence. The growing hydrogen industry puts pressure to develop new hydrogen sensors associated mainly with hydrogen mobility and energy storage. Cheap, reliable, and greenway syntheses are desired as well as well-performing materials which include applications not only to hydrogen storage and production but also to hydrogen sensing for security and/or performance monitoring.

Metal oxide semiconductors (MOSs) are the most used class of sensing materials due to their diverse composition, tunable bandgap properties, possible nanostructuring, and high chemical stability. They have been widely studied for being used as conductometric sensors, where the change of resistivity upon hydrogen presence was monitored [1,2]. Among MOSs, tungsten oxide (WO_3_) has attracted attention for the last few decades because of its interesting electrical, structural, and chemical properties [3,4]. Pure WO_3_ can easily detect oxidizing gases such as NO_2_, SO_2_, O_3_, etc. [5,6,7,8,9,10], but more challenging is an enhancement of its sensitivity towards reducing gases (H_2_, CO, NH_3_, etc.). This can be done by the loading of WO_3_ material by noble catalysts such as Au, Pt, and Pd [1,3,11,12,13]. 

Magnetron sputtering is a deposition technique that enables the microstructure to be controlled and the physical properties of the films to be tuned [14]. Reactive magnetron sputtering is popular because it is capable of producing compound films (including MOSs) with various stoichiometries and structures [15,16,17]. Among a wide variety of magnetron sputtering techniques, reactive high-power impulse magnetron sputtering (HiPIMS) is a prominent technique for the preparation of high-quality compound films. During the process, a reactive gas (e.g., O_2_) is dissociated, which improves its reactivity [18,19] and allows for the tuning of the stoichiometry and structure to be easier. Using HiPIMS, it is possible to alter the optical and electrical properties of thin films by varying the target power density [20]. At the same time, crystalline films can be synthesized at lower deposition temperatures compared to traditional sputtering techniques. Concerning the demands of applications to sensors, there are also a few disadvantages of HiPIMS, mainly the fact that most of the prepared films are dense and smooth.

Despite the aforementioned advantages, the preparation of tungsten oxide films by HiPIMS has rarely been reported [21,22]. In this work, we demonstrate a low-temperature (200 °C) synthesis of sub-stoichiometric WO_3−x_ films with a diverse structure by controlling the deposition parameters during reactive HiPIMS. We decorate the deposited films with Pd nanoparticles, which is a common way to increase the selectivity towards hydrogen; the catalytic behavior of Pd lowers the activation energy and thus the operating temperature range, as explained in the sensing mechanism [1,23,24]. We investigate and discuss the influence of the stoichiometry and structure on the hydrogen sensing performance of the prepared films in detail. The films are not subjected to high-temperature post-annealing and are annealed and stabilized only at the measurement temperature (below 350 °C) of the response of the sensor.

## 2. Experimental Section

### 2.1. Material Synthesis

Pd-WO_3−x_ films were prepared by a two-step deposition process onto 9 × 9 mm^2^ thermally oxidized Si substrates cleaned in an ultrasound bath in acetone, isopropyl alcohol, and deionized water for 10, 10, and 5 minutes, respectively.

The depositions were carried out in a cylindrical stainless-steel chamber (Z400, Leybold-Heraeus LH, Cologne, Germany, with a volume of 25 L and diameter of 42 cm, target-to-substrate distance of 70 mm) pumped by a turbomolecular pump (690 L/s for N_2_) supported by a scroll pump. The base pressure for all depositions was below 4 × 10^−3^ Pa.

In the first step, WO_3−x_ films were deposited using a metallic W target (99.95% purity) with a diameter of 72 mm connected to a high-power pulsed DC power supply (SIPP2000 USB, Melec, Baden-Baden, Germany) run at a fixed average power of 100 W with a pulse length in the range of 50–800 µs. The waveforms of the target voltage and current were recorded by a digital oscilloscope (TBS 2104, Tektronix Inc., Beaverton, OR, USA). A deposition temperature of 200 °C was kept constant for all depositions. Before the deposition, the pumping speed was adjusted (by a throttle valve) to achieve a pressure of 0.5 Pa at Ar flow rate of 15 sccm. Then, the oxygen gas was introduced as a reactive gas and its flow was automatically controlled to maintain a total pressure of 0.75 Pa.

One of the presented films was also deposited in the DC regime at the same average target power (100 W) but slightly changed the gas composition (i.e., O_2_:Ar was 1:2 for HiPIMS and 1:4 for DC). These are optimized conditions from the point of view of achieving a good crystallinity via DC sputtering at a substrate temperature of 200 °C.

The deposition rate was measured ex situ for each set of parameters by scanning a profile of a partially covered substrate by a profilometer. Then, the deposition time was adjusted to prepare films of a fixed thickness of 100 nm.

In the second step, the Pd deposition was carried out by using diode sputtering in RF mode at a power of 294 W (DC potential of 2.4 kV). The deposition time was 2 s, and the substrates were kept at room temperature.

### 2.2. Film Characterization

The morphology and topography of the films were examined by scanning electron microscopy (SEM; SU-70, Horiba Ltd., Kyoto, Japan) acquiring secondary electron image at 5 kV. The cross-sectional views of as-deposited films were used to verify the thickness of the films. For such imaging, the prescratched specimens were broken at ambient temperature. The aforementioned SEM system was used to investigate the elemental composition (stoichiometry) of the films by means of wavelength dispersive spectroscopy (WDS; Magnaray, Thermofisher Scientific, Waltham, MA, USA) done using standard reference samples of pure W and Fe_2_O_3_ (hematite) (Astimex Scientific Ltd.). Since it is difficult to precisely quantify the composition of thin films by employing WDS, thick films (thicker than 600 nm) were deposited deliberately to measure the composition of tungsten oxide films. These thicker films were deposited immediately after the deposition of the 100 nm thin films, while keeping the same deposition parameters.

The structure of the as-deposited films was analyzed by X-ray diffraction (XRD) using a diffractometer (X’Pert PRO, PANanalytical, Malvern, UK) with Cu K_α_ source of radiation in the Bragg–Brentano configuration with an ω-offset of 1.5°. The ω-offset was used to eliminate a strong reflection of the single-crystalline Si(100) substrate at 2θ angle of 69.17°. The Raman spectroscopy (LABRAM HR Evolution, Horiba Jobin Yvon, Palaiseau, France), using a 532 nm laser, was also employed to confirm the phase composition. 

A custom-built system was used to measure the gas response employing the four-point probe technique to calculate the change in the film electrical resistance in a time-varying gas mixture (H_2_ + synthetic air) and at a controlled temperature. For stable electrical contacts, four gold-plated spring-loaded pins with a 1 mm separation were pressed to the specimen surface placed in a cylindrical brass chamber (total volume of 3 cm^3^) heated by a hot plate. An independent thin thermocouple (0.3 mm thick) was pressed to the top surface to measure the temperature at the surface of the specimen. The flow of the gases was controlled by three separate mass flow controllers (Alicat Scientific Ltd., Tucson, AZ, USA). The resulting gas mixture was let to flow into the chamber with a total flow rate of 100 sccm. A DC source (KEITHLEY 6220, Keithley, Solon, OH, USA) and two electrometers (KEITHLEY 6514) were used for the electrical measurement. More details can be found in Ref. [25].

In this work, the sensitivity (*S*) at a specified concentration of H_2_ gas (*c*) and temperature (*T*) is defined as:

(1) $$S\left(c,\;T\right)=\frac{R_{a}}{R_{g}}$$

where *R*_a_ and *R*_g_ are the steady electrical resistances in the presence of synthetic air and H_2_ gas mixed with air, respectively. The values of *R*_a_ and *R*_g_ are obtained by fitting the resistance *R*(*t*) data by a sum of two exponential decays (in time *t*) with respect to Equation (2) in Reference [26].

## 3. Results and Discussion

To study the influence of the stoichiometry and structure of WO_3−x_ on the sensory response of Pd-WO_3−x_ films, a series of these films was prepared and studied. The presented results come from a set of specimens, where the average target power in a period during the deposition of tungsten oxide was kept constant and equal to 100 W, but the pulse length (*T*_on_) was varied from 50 to 800 μs. The duty cycle was also kept constant (1%), and therefore the average pulse target power density was 245.6 W/cm^2^ for all specimens. In addition to the discussion on the influence of the HiPIMS method, we also included one tungsten oxide film deposited in the DC regime. In this case, the average target power density was 2.46 W/cm^2^. From this point on, the films are referred to with respect to *T*_on_, e.g., “200 μs” film or “DC” film.

**Figure 1:**
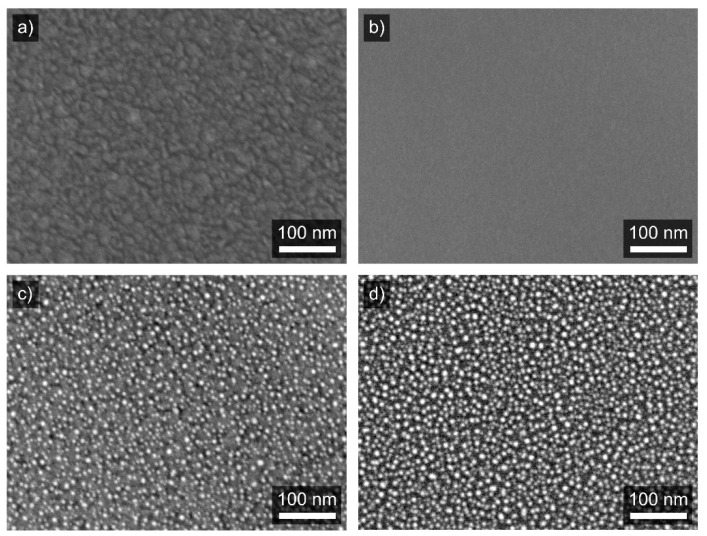
SEM micrographs of as-deposited (**a**,**b**) and Pd decorated tungsten oxide films after stabilization (**c**,**d**) for 100 µs and 800 µs films, respectively. It is clearly visible in (**c**,**d**) that the Pd particles in different films exhibit similar nature (shape and size).

After the deposition of all WO_3−*x*_ films in this series, a thin film (equivalent thickness lower than 1 nm) of Pd was deposited as a second deposition step. The coalescence of palladium on the surface occurs and Pd forms spherical nanoparticles. The appearance of the particles after stabilization of the films is shown in SEM micrographs in Figure 1**.** Despite the surface morphology of various films differing, we observed a comparable size of Pd particles formed by coalescence on all WO_3−x_. A discussion of the influence of the size of the Pd particles on the sensitivity of the films is out of the scope of this work. For further discussion, it is important only that the number of Pd particles varies in a range of approx. 60%, but the particles are similar in shape and size. See Supplementary Material containing an image analysis (Figure S1) and particle statistics (Figure S2) for the micrographs presented in Figure 1c,d. The changes are most probably connected to a different time of stabilization of different specimens. However, the same total amount of Pd material is guaranteed by its deposition onto all WO_3−*x*_ films at one run.

In the following sections, we describe the influence of the deposition parameters on the stoichiometry of the as-deposited films. Then, we compare the structure of the films in the as-deposited state with those which were stabilized during the sensory measurements at an elevated temperature (350 °C) by means of XRD and Raman spectroscopy. Finally, the hydrogen sensory response is presented and completed with a general discussion.

### 3.1. Deposition Parameters and Composition

**Figure 2:**
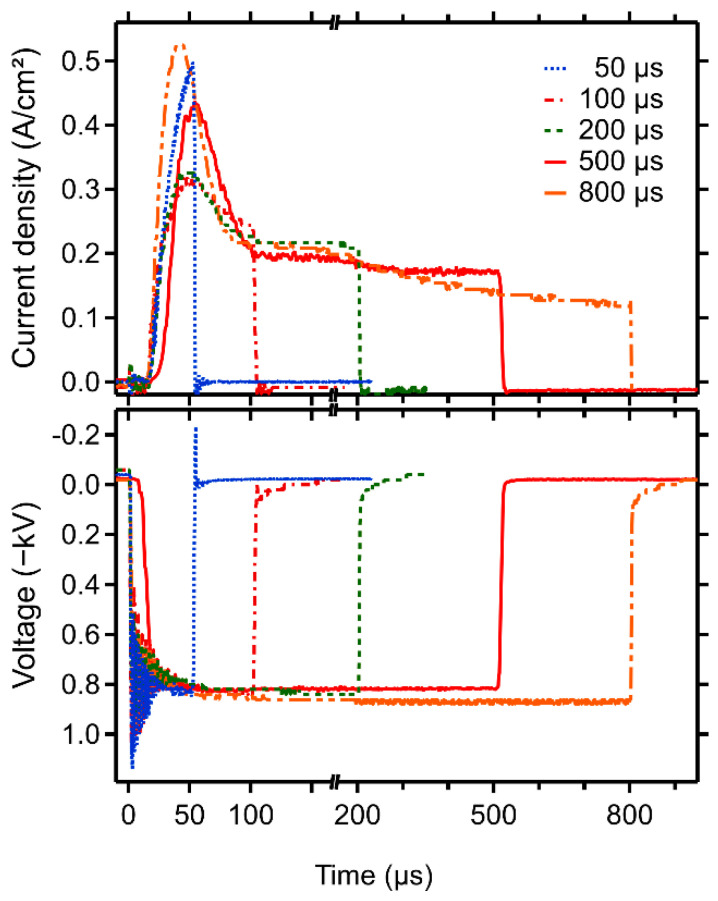
Voltage and current waveforms from deposition of high-power impulse magnetron sputtering (HiPIMS) films.

Target current density and voltage waveforms for different values of *T*_on_ are shown in Figure 2. The shape of the current density waveforms is typical for reactive HiPIMS under these discharge conditions: a current density peak in the beginning of the pulse and a gradual decrease of the current density towards the end of the pulse due to the rarefaction of the sputtering gas [27]. The peak values of the current density values are 0.55, 0.43, 0.32, 0.31, and 0.49 A/cm^−2^ at a 800, 500, 200, 100, and 50 µs pulse length, respectively. The unexpected high value and different shape of the current density for *T*_on_ = 50 µs can be explained by two effects. Mainly, the pulse ends after 50 µs, where other pulses achieve their maxima. Secondly, the deposition was carried out lastly after numerous experiments, and the target was more eroded, resulting in a stronger magnetic field and thus increasing plasma conductivity.

**Figure 3:**
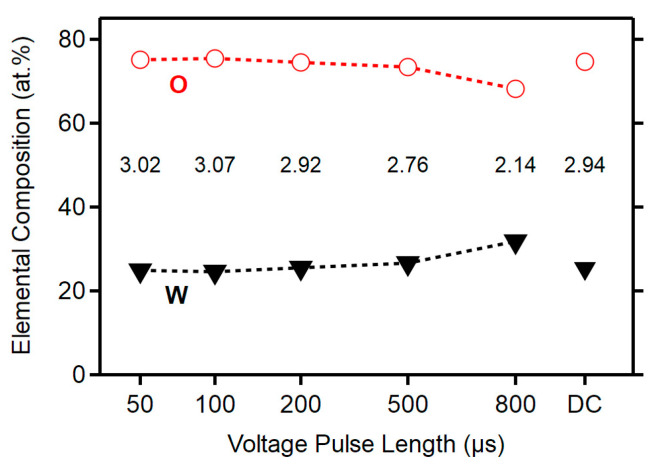
Elemental composition of films and stoichiometry. The stoichiometry ([W]/[O] ratio) decreases with an increasing voltage pulse length; the DC film is slightly sub-stoichiometric.

The effect of the HiPIMS parameters on the elemental composition of the deposited films is demonstrated in Figure 3, where WDS results are shown. The stoichiometry of the films calculated as a ratio of the atomic concentration of oxygen and tungsten is displayed in the tags in Figure 3 as well. A decrease in the oxygen concentration with increasing *T*_on_ from 50 to 800 µs was observed. In reactive HiPIMS, there is target poisoning that is crucial at a shorter *T*_on_. It means that the reactive gas (O_2_) easily produces a compound layer on the target surface and the sputtering yield decreases. This affects the amount of oxygen in the deposited material, which results in changes in the stoichiometry of the films. However, the target poisoning is moderate at higher pulse lengths, which reduces the thickness of the compound layer and produces an excessive amount of metal ions [28,29].

### 3.2. Structure

**Figure 4:**
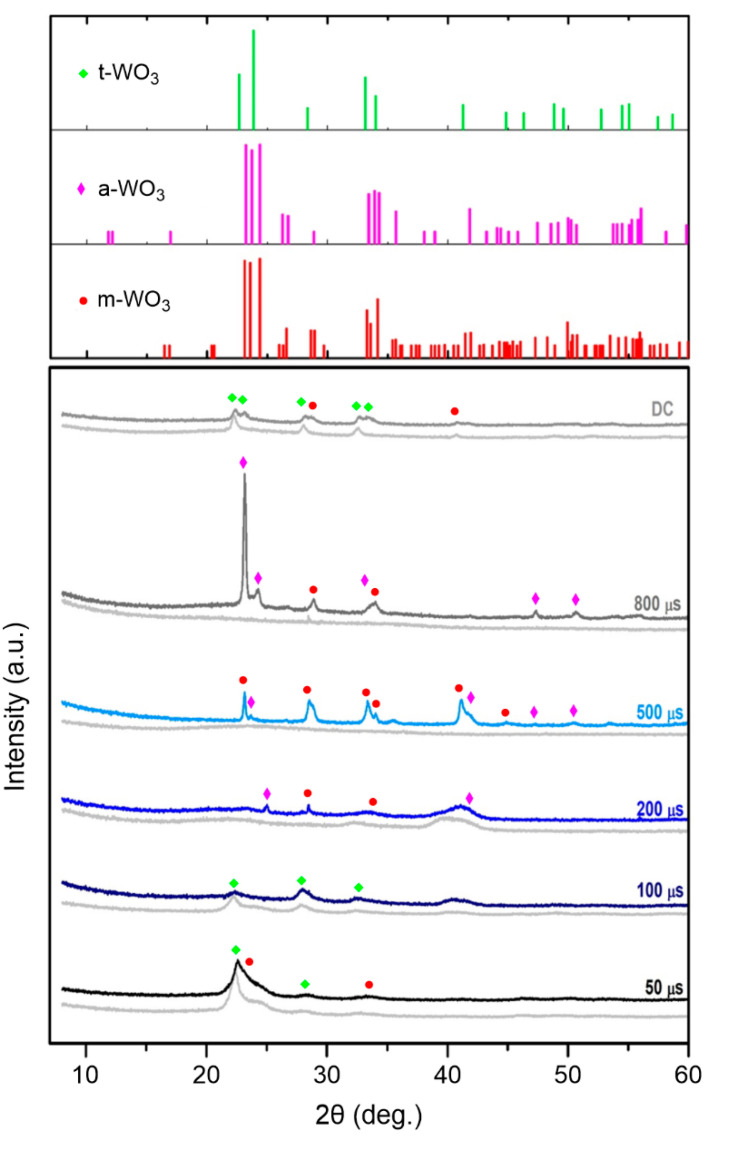
XRD patterns of as-deposited films (light grey) and annealed/stabilized films (color traces) after sensory measurements at 350 °C.

A comparison of XRD patterns of as-deposited films and annealed films (stabilized during the sensory measurements at 350 °C) is shown in Figure 4. As for the deposited films (grey traces), 500 µs and 800 µs films are found to be amorphous, while 50 µs, 100 µs, 200 µs, and DC films are crystalline or nanocrystalline. Due to the overlapping of relatively broad diffraction peaks, it is, however, unfavorable to unambiguously determine a dominant phase in each film. Only the pattern of DC film corresponds well to that of the tetragonal WO_3_ phase (PDF Card No. 04-007-0954). In the case of 50 µs and 100 µs films, it seems that the major phase is tetragonal, but the presence of triclinic/anorthic (PDF Card No. 01-073-6498) and monoclinic (PDF Card No. 01-083-0950) phases cannot be unambiguously excluded (see the powder diffraction standards in the figure).

During measuring the sensory response to hydrogen (mixed with ambient air) at 350 °C, the crystalline/nanocrystalline structure of some films is changed (color patterns in Figure 4). The structure of the 50 µs and 100 µs films does not change significantly (very small changes in the pattern for the 50 µs film can imply the occurrence of the monoclinic phase). The relatively high stability of their structure can be attributed to their stoichiometry close to 3.0, i.e., no significant reaction with ambient oxygen occurs. Similar situation is also observed for the 200 µs film, where no pronounced changes in the structure are detected, and only a few crystallites of triclinic and/or monoclinic phase are formed. The situation is completely different in the case of the 500 µs and 800 µs films with the amorphous structure and low stoichiometry in the as-deposited state. These films in the as-deposited state exhibit the stoichiometry of 2.76 and 2.14, respectively. A possible reaction with ambient oxygen results in a crystallization and stabilization of their structure with a dominant monoclinic WO_3_ phase in the case of 500 µs film and a triclinic one in the case of 800 µs film. The patterns are very close to each other and the presence of both phases in each film cannot also be ruled out only based on XRD. In the case of the DC film, new diffraction peaks corresponding to the monoclinic phase appear, and this phase thus coexists with the tetragonal one after stabilization. It should be noted that no phases corresponding to the presence of Pd particles on the surface were detected.

**Figure 5:**
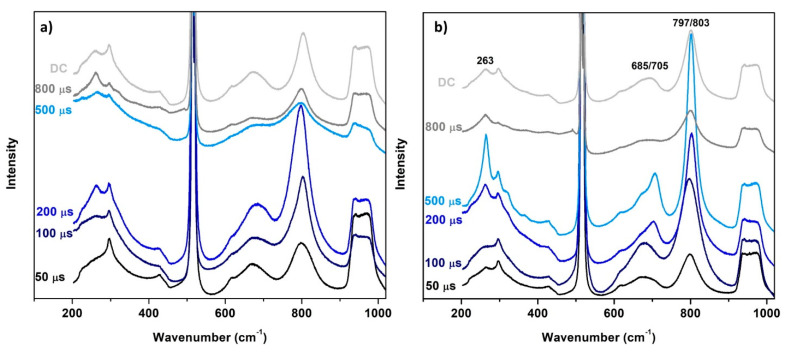
Raman spectra of (**a**) as-deposited films and (**b**) annealed films after sensory measurements at 350 °C.

The findings of XRD are supported by the results of Raman spectroscopy. The spectra of the as-deposited and annealed films are shown in Figure 5. Raman peaks at 263, 322, and 368 cm^−1^ are attributed to the deformation vibrations of W–O bonds, while the bands at 685, 704, and 797–803 cm^−1^ are the characteristics of stretching modes of O–W–O bonds [30,31,32]. Other features visible in the spectra are to be attributed to silicon substrate (mainly Si–Si peaks at 301, 616, 670 cm^−1^ and a broad feature at 935–975 cm^−1^; Si–O peaks at 428 and 950 cm^−1^).

The peaks appearing at approx. 685 and 797 cm^−1^ can imply several phases such as triclinic, tetragonal, or orthorhombic and therefore cannot be used to distinguish among them. However, the peak observed at 704 cm^−1^ in annealed 200 µs, 500 µs, and DC films corresponds exclusively to the monoclinic phase [32,33,34]. The monoclinic phase is also indicated by the shift of the peak at 797 cm^−1^ towards 803 cm^−1^, which can also be observed for the 200 µs and 500 µs films. The spectrum of the annealed 500 µs film also shows peaks at 322 and 368 cm^−1^ which can be attributed to lower stretching modes of the triclinic phase [31,35]. These two peaks are also visible in the spectrum of the 800 µs film.

### 3.3. Sensory Response and Discussion

**Figure 6:**
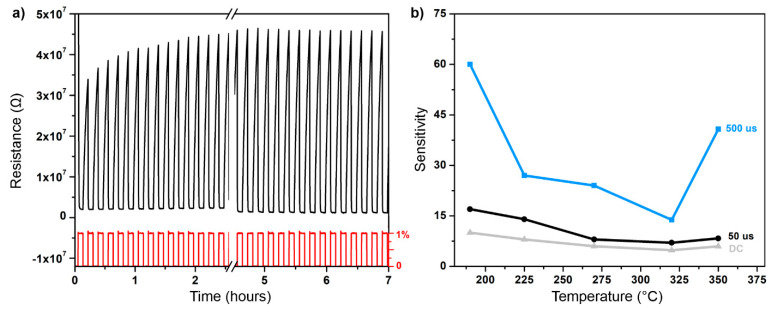
(**a**) Stabilization of the 500 µs film at 350 °C. The hydrogen in the concentration of 1% was cycled with a period of 5 min until the response was stable after approx. 5 h. (**b**) Sensitivity to 1% of H_2_ for selected films at various temperatures.

To characterize the sensory characteristics of the prepared films, the specimens were assembled as conductometric gas sensors and exposed to various concentrations of hydrogen gas in synthetic air at various temperatures. Firstly, the films were kept and measured at the highest examined temperature (350 °C) until the response was stable. This was demonstrated for the 500 µs film in Figure 6a where the response is stabilized after approx. 5 h (reaching 99% of the steady value) of the cycling of clean air and 1% of hydrogen. After the stabilization, the lower working temperatures were tested. The dependency of the sensitivity of selected films on working temperature is plotted in Figure 6b. The highest stable response was achieved at around 190 °C for all films. For the temperature at 350 °C, there is a visible increase of sensitivity for all films, which can be attributed to the onset of the thermal dissociation of hydrogen. The optimal temperatures (with the highest response) for particular specimens vary slightly but do not differ much from 190 °C. Therefore, it is possible to compare the response of the investigated films at 190 °C, where the response approaches the maximum values for all investigated films.

**Figure 7:**
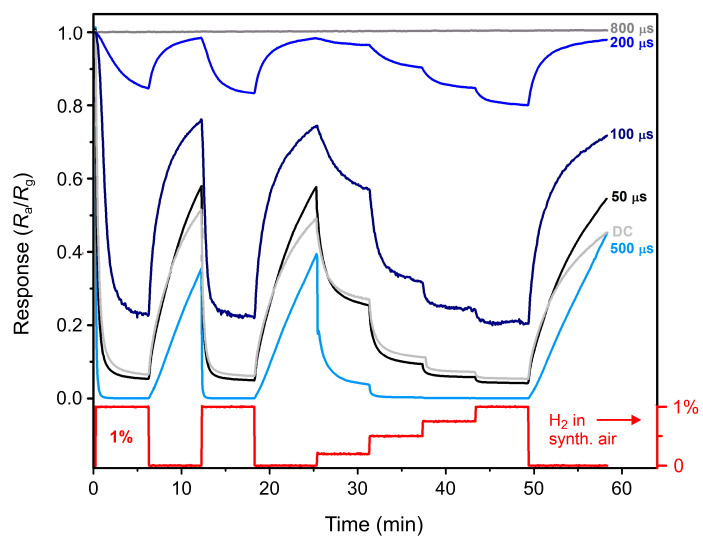
Relative response of all investigated films to the sequence of various hydrogen concentrations.

A time dependence of the relative response ($R_{a}/R_{g}$) to a sequence of various hydrogen concentrations at a temperature of 190 °C is displayed in Figure 7. Here, one can compare the response of all films to the same amount of hydrogen. The highest response is plotted for 500 µs, 50 µs, and DC films; 100 µs and 200 µs films exhibit lower responses, and 800 µs film does not respond to hydrogen at all.

The presented data in Figure 7 corresponding to expositions to 1% of hydrogen were fitted with a sum of two exponential decays [26], and the sensitivity was calculated according to Equation (1). The obtained numerical values are summed up in Table 1.

The largest sensitivity is found for the 500 µs film, which exhibits almost pure monoclinic structure. The other two well-performing films (50 µs and DC) also show an evidence of the presence of the monoclinic phase, but the major phase remains tetragonal. One can identify a trend that the more crystalline is the film (and the more monoclinic), the higher sensitivity is achieved. This trend is also corroborated by poorly responding 100 µs and 200 µs films, whose crystallinity is low. Finally, the 800 µs film is well crystalline but of a very high conductivity. A possible explanation is that only the upper part of the material (most probably the very surface) oxidizes into crystalline trioxide. The rest of the material remains sub-stoichiometric and so with a high conductivity. Since the material is measured as a thin film, the total resistance is low, and the trioxide portion of the material does not affect the resistance and so the sensitivity of the film considerably.

The findings concerning crystalline phases are in good agreement with the works in References. [36,37], where different crystalline structures of tungsten trioxide lead to considerably different responses. Here, the advantage is that the films were prepared just by a small variation of the power supply settings, which ends up in a possibility to control the stoichiometry.

One may also correlate the sensory response with the electrical resistance of the films. In Table 1, it is seen that the best-performing films are those with the highest resistance. A variation of resistance in oxides such as WO_3_ is strongly connected with the concentration of oxygen vacancies. It has been shown that the oxygen vacancies play an important role in enhancing the performance of tungsten oxide material based sensors [38,39]. Nevertheless, the amount of oxygen vacancies must not prevent the proper crystallization, which is also important for the sensory response.

In our case, it was impossible to prepare the monoclinic phase directly in the as-deposited state. However, the prepared stoichiometry ([O]/[W] = 2.76 ± 0.09) of the 500 µs film was shown to be appropriate for providing a possibility to evolve the monoclinic structure during the stabilization and oxidization of the film at 350 °C in air and thus to form a film with a high resistance. In contrast, the 800 µs film was too sub-stoichiometric (2.15 ± 0.09) to reform completely into a trioxide crystalline film. The over-stoichiometry (3.07 ± 0.09) of the 100 µs film prevented it from transforming the crystalline structure at a relatively low annealing temperature. The stoichiometry (3.01 ± 0.09) of the 50 µs film and the light sub-stoichiometry (2.94 ± 0.09) of DC film provided a possibility for the formation of the monoclinic phase, but still the major phase remained tetragonal (see Figure 4). In the case of the 200 µs film, there is probably only a small portion of the material that can be reformed to the monoclinic phase, so the material responds to hydrogen, but not ideally. 

Since the amount of the Pd particles and their shape are similar for all films, we deliberately exclude it from the discussion on the differences in the sensory behavior of individual films. However, it is indisputable and should not be forgotten that the catalytic effect of palladium is crucial for the sensory response of the films (Pd-free films do not sense, not shown), and Pd is fully responsible for lowering of the working temperature.

The focus of this work was to present a possibility of preparing hydrogen sensing films using the benefits of an environmentally friendly magnetron sputtering technique such as HiPIMS at low temperature. To conclude the discussion, we append Table 2 including similar architectures combining tungsten trioxide and noble metals (Pd or Pt) published in the literature. Most of the works have similar values of sensitivity but, for a few times, a lower hydrogen concentration. However, the sensitivity cannot be evaluated as a single parameter, and the table is presented to show that the response of our films is in a similar order. It should be noted that all of these materials were prepared mostly by wet processes such as sol-gel [40], screen-printing [3], etc. As one can see, the sensitivity of our best films is comparable to those that were usually prepared at higher temperatures. In our case, it was not necessary to sinter and/or anneal the material at higher temperatures and the best results were achieved when the specimens were deposited at 200 °C and stabilized at 350 °C. These conditions are closer to the application of hydrogen sensing films on temperature-sensitive substrates such as plastics.

## 4. Conclusions

In this work, we demonstrated the suitability of a low-temperature (200 °C) synthesis of WO_3−x_ films by using the reactive high-power impulse magnetron sputtering technique. By changing the pulse length only, we were able to prepare the films with various stoichiometry and structure. Subsequently, the WO_3−x_ films were decorated by Pd particles and stabilized at 350 °C in air.

We showed that the stoichiometry affects not only the as-deposited structure but plays an important role in the structure of the stabilized films. While the crystalline or nanocrystalline films with the stoichiometry close to 3.0 are relatively thermally stable without significant changes in the structure during the stabilization, the amorphous films with the higher level of the sub-stoichiometry further oxidize and form well crystalline structures during the stabilization. The type of WO_3_ phase is then a key factor determining the performance of the sensor to hydrogen. Particularly, the film with a stoichiometry of 2.76 ± 0.09 deposited under proper conditions by reactive HiPIMS crystallizes into the monoclinic structure during the stabilization at 350 °C in air and exhibits enhanced sensory ability to hydrogen than other crystalline phases (higher sensitivity).

A further development of this synthesis approach would lead to the application of tungsten trioxide based thin films on various substrates (such as plastics), which cannot withstand common techniques of synthesis at high temperatures. A further improvement of a direct deposition of the sensing film with the monoclinic WO_3_ phase is desirable.

## Figures and Tables

**Table 1:** Important properties of the prepared films sorted by the acquired sensitivity to 1% of hydrogen. The stoichiometry of as-deposited films [O]/[W] is to be considered with an absolute error of 0.09 (based on the estimated errors of EDS analysis). The sheet resistance was measured at 190 °C in synthetic air.

Film	Sensitivity $R_{a}/R_{g}$	Crystallinity after Annealing	Crystalline Phases	Sheet Resist. (Ω/sq)	As-Deposited Stoichiometry	Response Time (s)
500 µs	60	moderate	monoclinic (triclinic)	3.4 × 10^8^	2.76	10
50 µs	17	moderate	tetragonal + monoclinic	7.3 × 10^6^	3.01	38
DC	10	moderate	tetragonal + monoclinic	1.2 × 10^6^	2.94	70
100 µs	3.8	low	tetragonal + triclinic	1.7 × 10^5^	3.07	113
200 µs	2.0	low	triclinic (monoclinic)	2.0 × 10^5^	2.92	106
800 µs	–	high	triclinic (monoclinic)	6.3 × 10^2^	2.15	–

**Table 2:** Comparison table of published materials combining tungsten trioxide with palladium or platinum. The appropriate definitions of sensitivity are noted for individual works.

Material	Response	Sensitivity Definition	Temperature, °C	Concentration, ppm	References
WO_3_ thin film	5	*S* =*R*_a_/*R*_g_	150	250	[40]
Pd-WO_3_ nanocomposites	19	*S* = (*R*_a_ − *R*_g_)/*R*_g_	200	200	[41]
Pt-WO_3_ thin film	~5	*S* = (*R*_a_ − *R*_g_)/*R*_g_	400	500	[42]
Pd-Graphene QDs on WO_3_ thin film	1.12	*S* = (*R*_a_ − *R*_g_)/*R*_g_	150	3600	[4]
Pt-WO_3_ nanowires	20	*S* = *R*_a_/*R*_g_	200	100	[43]
Pd-WO_3_ nanolamellae	69	*S* = (*R*_a_ − *R*_g_)/*R*_g_	180	200	[3]
Pd-WO_3_	12	*S* = *R*_a_/*R*_g_	190	2000	This work
Pd-WO_3_	60	*S* = *R*_a_/*R*_g_	190	10,000	This work

## Supplementary Materials

The following are available online at https://www.mdpi.com/1996-1944/13/22/5101/s1, Figure S1: Binarized SEM micrograph. The Pd particles were masked by Otsu’s method. Figure S2: Histogram of equivalent radius of Pd particles gained from SEM image analysis.
