# Supplementary material for: Tuning Stoichiometry and Structure of Pd-WO3−x Thin Films for Hydrogen Gas Sensing by High-Power Impulse Magnetron Sputtering
Source: Materials (Basel). 2020 Nov 12;13(22):5101. doi: 10.3390/ma13225101 (PMC7697909; doi:10.3390/ma13225101)
Supplement: Supplementary file 1 [file materials-13-05101-s001.pdf]

materials

Supplementary: Tuning Stoichiometry and Structure of Pd-WO3-x Thin Films for Hydrogen Gas Sensing by High-Power Impulse Magnetron Sputtering

Nirmal Kumar, Stanislav Haviar*, Jiří Rezek, Pavel Baroch and, Petr Zeman

Department of Physics and NTIS – European Centre of Excellence, Faculty of Applied Sciences, University of West Bohemia, Univerzitní 8, 306 14 Plzeň, Czech Republic; kumarn@kfy.zcu.cz (N.K.); jrezek@ntis.zcu.cz (J.R.); pbaroch@kfy.zcu.cz (P.B.); zemanp@kfy.zcu.cz (P.Z.)

* Correspondence: haviar@ntis.zcu.cz

1. Analysis of Pd Particles from SEM Micrographs

The SEM micrographs were optimized for contrast, binarized (by Otsu's method) and the particles were statistically evaluated. The particles are considered to have a circular cross section, so the corresponding disc radius was chosen as a comparative parameter.

The figure at the side contains an example of the binarization of the image (highlighting particles):

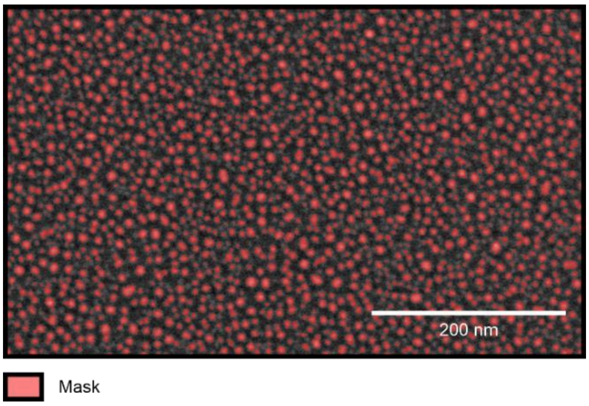

Here two histograms of particle radii gained from two micrographs publish in the article are presented:

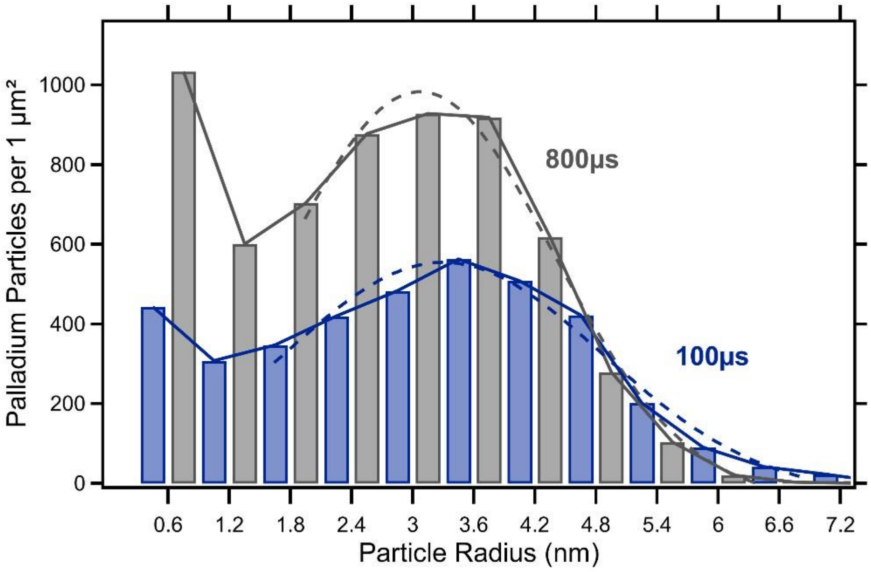

The two specimens differ in the amount of particles, but the sizes are quite similar. There is a family of small particles with a diameter below resolution of the microscope of approx. 1 nm. A second family of larger particles can be analyzed, where the radius given by Gaussian fit is 3.5 and 2.9 nm for 100µs and 800µs, respectively. The widths of these distributions are 2.2 and 1.8 nm, respectively.

Supplementary material: Tuning Stoichiometry and Structure of Pd WO_{3-x} Thin Films for Hydrogen Gas Sensing by High Power Impulse Magnetron Sputtering, *Kumar et al.*
